# Randomized phase II study of three doses of the integrin inhibitor cilengitide versus docetaxel as second-line treatment for patients with advanced non-small-cell lung cancer

**DOI:** 10.1007/s10637-012-9842-6

**Published:** 2012-07-03

**Authors:** Christian Manegold, Johan Vansteenkiste, Felipe Cardenal, Wolfgang Schuette, Penella J. Woll, Ernst Ulsperger, Anne Kerber, Josef Eckmayr, Joachim von Pawel

**Affiliations:** 1 Medizinische Fakultät Mannheim der Universität Heidelberg, Theodor-Kutzer-Ufer 1-3, 68167 Mannheim, Germany; 2 University Hospital Gasthuisberg, Herestraat 49, 3000 Leuven, Belgium; 3 Institut Català d’Oncologia, Avgda Gran Via 199-203, 08907 L’Hospitalet, Barcelona Spain; 4 Krankenhaus Martha-Maria Halle-Dölau, Roentgenstraße 1, 06120 Halle, Germany; 5 University of Sheffield, Whitham Road, S10 2SJ Sheffield, UK; 6 KHR Hietzing, Wolkersbergenstraße 1, 1130 Vienna, Austria; 7 Merck KGaA, Frankfurter Straße 250, 64293 Darmstadt, Germany; 8 Klinikum Kreuzschwestern Wels, Grieskirchner Straße 42, 4600 Wels, Austria; 9 Asklepios Fachkliniken München-Gauting, Robert-Koch-Allee 2, 82131 Gauting, Germany; 10 Chirurgische Universitätsklinik – Interdisziplinäre Thorakale Onkologie, Universitätsmedizin, Medizinische Fakultät Mannheim der Universität Heidelberg, Theodor-Kutzer-Ufer 1-3, 68167 Mannheim, Germany

**Keywords:** Cilengitide, Integrin inhibitor, Docetaxel, Non-small-cell lung cancer, Second-line treatment

## Abstract

*Introduction* This multicenter, open-label, phase II study was carried out to compare the efficacy and safety of cilengitide (EMD 121974), a selective inhibitor of the cell-surface integrins αVβ3 and αVβ5, with that of docetaxel in patients with advanced non-small-cell lung cancer (NSCLC). *Methods* Patients (*n* = 140) with advanced NSCLC who had failed first-line chemotherapy were randomized to cilengitide 240, 400, or 600 mg/m^2^ twice weekly, or docetaxel 75 mg/m^2^ once every 3 weeks for eight cycles. Non-progressing patients could continue cilengitide for up to 1 year. The primary endpoint was progression-free survival (PFS). No statistical tests were performed since the study was exploratory in nature and the number of patients enrolled was relatively small. *Results* Median PFS was 54, 63, 63, and 67 days for cilengitide 240, 400, and 600 mg/m^2^, and docetaxel 75 mg/m^2^, respectively. One-year survival rates were 13 %, 13 %, 29 %, and 27 %, respectively. The response rate (partial response only) with docetaxel was 15 %. No responses were reported in any cilengitide arm. The most frequent grade 3/4 treatment-related adverse events in the docetaxel group were leukopenia and neutropenia (experienced by 13 % of patients). Hematologic toxicity of this severity did not occur in cilengitide-treated patients. *Conclusion* With the highest dose of cilengitide (600 mg/m^2^), median PFS and 1-year survival were similar to those in patients treated with docetaxel 75 mg/m^2^ and there were fewer grade 3/4 treatment-related adverse events.

## Introduction

Lung cancer is the leading cause of cancer-related death worldwide [1], and the majority of cases (85–90 %) are non-small-cell lung cancer (NSCLC) [2]. Only 30–35 % of patients with NSCLC present with sufficiently localized disease at diagnosis to attempt curative surgical resection (stages IA and IB, IIA and IIB, and IIIA), and ~50 % of those who undergo surgical resection will experience local or systemic relapse. Thus ~80 % of all patients with lung cancer are considered for chemotherapy at some point during the course of their illness, and platinum-based combination regimens are still considered standard of care for the majority of patients in the first-line treatment of NSCLC [3].

At the time of this study (1999–2001), single-agent docetaxel (75 mg/m^2^ every 3 weeks) was the only approved chemotherapy for the second-line treatment of advanced NSCLC in the USA and Europe, having demonstrated longer survival and better quality of life than best supportive care alone [4, 5], and higher rates of 1-year survival than vinorelbine or ifosfamide [6]. Since then, although second-line options have broadened, efforts to improve on the efficacy of docetaxel, either by using an alternative agent or by combining docetaxel with carboplatin, have not proved successful [7, 8], and single-agent docetaxel remains one of the second-line standards of care. However, docetaxel has only moderate activity and is associated with non-hematologic and hematologic toxicity, in particular, neutropenia and febrile neutropenia [4, 6]. There is therefore a continuing need for new therapies which are active in NSCLC and which have a favorable safety profile giving them potential for use in combination.

Cilengitide (EMD 121974) is the first compound in a new class of targeted anticancer therapies – the integrin inhibitors – to reach phase III clinical trial, and is currently in phase III in glioblastoma. Integrins are heterodimeric transmembrane receptors that play key roles in the interaction between cells, adhesion to the extracellular environment, and cell migration involved in angiogenesis and tumor development [9–12]. Cilengitide is a novel cyclized arginine–glycine–aspartic acid-containing pentapeptide that selectively inhibits the cell-surface integrins αVβ3 and αVβ5. Cilengitide is thought to act on αVβ3- and αVβ5-expressing tumor cells both directly, by deactivating signals involved in survival and growth, and indirectly, by inhibiting angiogenesis and thereby tumor growth. It blocks endothelial and tumor-cell adhesion, migration, and differentiation [13–16].

The current clinical development program for cilengitide includes glioblastoma (the phase III CENTRIC and phase II CORE trials), head and neck cancer (phase II, ADVANTAGE), and NSCLC (phase II, CERTO). The randomized phase II trial in the first-line NSCLC setting (clinicaltrials.gov NCT00842712) is investigating the effects of adding cilengitide to cetuximab and platinum-based chemotherapy. Here we report the first phase II study (EMD 121974–003) to examine the effects of cilengitide in patients with NSCLC. It investigated the efficacy and safety of three different doses of single-agent cilengitide compared with docetaxel monotherapy as second-line treatment in patients with advanced NSCLC who had failed first-line chemotherapy.

This study was conducted in the period 1999–2001 and its results were not published – in fact, the further clinical development of cilengitide was put on hold. However, the growing interest in anti-angiogenesis as a therapeutic approach prompted renewed interest in the molecule and has resulted in the extensive trial program noted above. In 2009, the results of this early NSCLC trial were published in poster form [17], and attracted interest. Moreover, this study remains relevant since the comparator used, docetaxel, is still a standard of care in second-line NSCLC, and efforts continue to find a drug with which to combine it without adding unacceptable toxicity. This study provides clinical evidence for the activity of cilengitide in NSCLC, complementing the preclinical in vivo data suggesting antitumor efficacy and evidence from tumor tissue that integrin overexpression may be of prognostic significance in NSCLC [18].

## Materials and methods

### Study design

This was a multinational, multicenter, open-label, randomized, parallel-group, phase II study in patients with advanced (stage IIIB and IV) NSCLC who had failed first-line chemotherapy. Patients were randomized to one of four treatment groups: cilengitide 240, 400, or 600 mg/m^2^ twice weekly, or docetaxel 75 mg/m^2^ once every 3 weeks for eight cycles. The initial treatment period was 6 months, but patients whose tumor growth was controlled by cilengitide could continue therapy for an additional 6 months, to a maximum treatment period of 1 year. On completion or withdrawal from the study, all patients were followed until the end of the study to assess overall survival (OS).

The primary endpoint was progression-free survival (PFS). Secondary endpoints were response rate, OS, safety, and tolerability.

### Patients

Both genders were eligible for inclusion, but women had to be postmenopausal or infertile. Other eligibility criteria were: (i) age ≥18 years (≥19 years in Austria); (ii) cytologically or histologically confirmed advanced NSCLC; (iii) at least one measurable/evaluable lesion according to Response Evaluation Criteria In Solid Tumors (RECIST) not located within the area of any previous radiation; (iv) failure of first-line therapy (defined as disease progression during first-line therapy or after its discontinuation); (v) life expectancy ≥12 weeks; (vi) only one previous chemotherapy regimen (one to six courses); (vii) Karnofsky performance status ≥70 %; and (viii) adequate renal function (creatinine <2 × upper limit of normal, ULN), hepatic function (total bilirubin <2 × ULN and serum transaminases ≤3 × ULN, or ≤5 × ULN in patients with known liver metastases), and hematologic function (granulocytes ≥1500/mm^3^, platelets ≥100,000/mm^3^, and hemoglobin ≥10 g/dL).

Exclusion criteria were: (i) pregnancy or breast-feeding; (ii) chemotherapy and/or radiotherapy treatment or major surgery within 4 weeks of study entry; (iii) previous treatment with docetaxel or anti-angiogenic therapy; (iv) history of brain metastases; (v) history of cerebrovascular accident or transient ischemic attack; clinically significant cardiac or cardiovascular abnormalities (New York Heart Association III/IV), or unstable angina or arrhythmias (Lown grading system for cardiac arrhythmias grade IV) requiring treatment; (vi) bypass surgery within 6 months prior to treatment; (vii) known active infection with human immunodeficiency virus, hepatitis B virus, or hepatitis C virus; or (viii) history of paclitaxel or docetaxel allergy.

The protocol was approved before the start of the study by the relevant Independent Ethics Committees in the participating institutions. The study was performed in accordance with the Declaration of Helsinki, Good Clinical Practice guidelines, and applicable regulatory requirements. Written informed consent was obtained from all patients at the screening visit.

### Treatment

Patients were randomized to one of three cilengitide doses (240, 400, or 600 mg/m^2^) twice weekly as a 1-hour intravenous infusion or docetaxel 75 mg/m^2^ as a 1-hour intravenous infusion once per cycle over 8 cycles. A cycle was defined as a treatment period of 3 weeks. Steroid prophylaxis was given to patients in the docetaxel group. The doses of cilengitide chosen for study were based on experience in a phase I trial in which the amount of drug administered was escalated from 30 mg/m^2^ to 600 mg/m^2^ without encountering unacceptable toxicity. In the absence of a defined maximum tolerated dose for cilengitide, the three highest dose levels used in phase I (ie, 240, 400, and 600 mg/m^2^) were chosen for comparison with docetaxel in this randomized phase II trial.

### Assessments

PFS was defined as the time interval between the date of randomization and the date of disease progression or death, whichever occurred first. Tumor response was assessed according to the first version of RECIST [19], based on the size of the target lesions, as determined by computed tomography and/or magnetic resonance imaging. Response rate was defined as the sum of the rates of complete response (CR) and partial response (PR) per treatment arm. Tumor growth control was defined as the sum of the CR, PR, and stable disease (SD) rates. OS was defined as the time from the start of study drug administration until death. Safety was evaluated by laboratory assessments, physical measurements (heart rate; systolic/diastolic blood pressure; body temperature; body weight; status according to the Karnofsky Performance Index; physical examination electrocardiogram [ECG]), and monitoring of adverse events (AEs) which were graded according to the National Cancer Institute (NCI) Common Toxicity Criteria (CTC) system version 2. The AE data were re-analyzed in March 2011 using MedDRA® Terminology Version 10.0.

### Statistical analysis

Given the relatively small number of patients in each treatment arm and the exploratory nature of the trial, it was not intended that tests of statistical significance (or subgroup analyses) would be undertaken. The study was not powered to demonstrate non-inferiority. Results were interpreted with descriptive statistics using SAS version 8.

The primary efficacy analysis was performed on an intention-to-treat (ITT) basis, including all randomized patients, and a Kaplan–Meier estimate of survival probabilities was constructed. The safety analyses included all randomized patients who had taken at least one dose of a study drug.

## Results

### Patients

In total, 140 patients from 30 centers in seven countries (Austria, Belgium, France, Germany, the Netherlands, Spain, and the UK) were randomized and included in the study (ITT analysis). The first patient was enrolled in November 1999 and the study was completed in October 2001. Three patients did not receive any study medication (one in the cilengitide 240 mg/m^2^ group and two in the docetaxel group), meaning that 137 patients were included in the safety analysis. Patient baseline characteristics are shown in Table 1: the majority was male (67 %), their mean age was 60.2 years (range 33–80 years), and 96 % had stage IV disease. Given the date of the study, histology data were not documented as rigorously as would be the case in current trials (and 46 % of tumors were not specified in this regard). However, from the data available, the predominant histology across treatment groups was adenocarcinoma. The majority of patients had received platinum-based regimens as first-line therapy. The proportion of patients who had had tumor-related surgery was higher among patients randomized to docetaxel and 600 mg/m^2^ cilengitide than in those randomized to lower doses of the integrin inhibitor. Overall, however, there were no clinically important differences between the treatment groups with regard to baseline characteristics.

**Table 1:** Baseline characteristics of intention-to-treat population

Characteristics	Cilengitide 240 mg/m^2^ *n* = 35	Cilengitide 400 mg/m^2^ *n* = 35	Cilengitide 600 mg/m^2^ *n* = 36	Docetaxel 75 mg/m^2^ *n* = 34	Total *n* = 140
Male/female, *n* (%)	25/10 (71/29)	25/10 (71/29)	22/14 (61/39)	22/12 (65/35)	94/46 (67/33)
Mean age (range), yrs	62.5 (45.0–80.0)	57.8 (33.0–77.0)	59.3 (41.0–76.0)	61.2 (42.0–79.0)	60.2 (33.0–80.0)
Karnofsky PS *n* (%)					
100 %	4 (11)	5 (14)	5 (14)	5 (15)	19 (14)
90 %	8 (23)	14 (40)	12 (33)	10 (29)	44 (31)
80 %	17 (49)	12 (34)	12 (33)	12 (35)	53 (38)
70 %	6 (17)	4 (11)	7 (19)	7 (21)	24 (17)
Tumor stage, *n* (%)					
IIIB	1 (3)	2 (6)	0	3 (9)	6 (4)
IV	34 (97)	33 (94)	36 (100)	31 (91)	134 (96)
Histology^a^, *n* (%)					
Adenocarcinoma	14 (40)	12 (34)	12 (33)	9 (26)	47 (34)
Squamous	2 (6)	4 (12)	4 (12)	8 (24)	18 (13)
Other	4 (11)	2 (6)	2 (6)	2 (6)	10 (7)
Unknown	15 (43)	17 (49)	18 (50)	15 (44)	65 (46)
Prior chemotherapy, *n* (%)					
Platinum based	25 (71)	25 (71)	28 (78)	23 (68)	101 (72)
Non-platinum	10 (29)	10 (29)	8 (22)	11 (32)	39 (28)
Prior radiation, *n* (%)	12 (34)	12 (34)	11 (31)	15 (44)	50 (36)
Tumor-related surgery, *n* (%)	6 (17)	8 (23)	14 (39)	12 (35)	40 (29)

^a^Given the date of the study, histology was not performed as rigorously as would be the case in current trials

Karnofsky PS, Karnofsky performance status

### Treatment

Median (range) duration of treatment was 41 (4–165), 50 (15–155), and 60 (1–253) days with cilengitide 240, 400, and 600 mg/m^2^, respectively, and 48 (1–163) days with docetaxel. The main reason for study discontinuation was progressive disease (93 patients, 66 %), which occurred more frequently with cilengitide (71 % of patients) than docetaxel (50 %). There were no important differences between the treatment groups with respect to the percentage of patients who withdrew due to AEs (~14 % in the cilengitide groups vs ~12 % in the docetaxel group).

Eight patients (6 %) completed eight cycles of treatment: one patient (3 %) in the cilengitide 240 mg/m^2^ group, two patients (6 %) in the 600 mg/m^2^ group, and five patients (15 %) in the docetaxel group. One patient (3 %) in the cilengitide 600 mg/m^2^ group completed 11 cycles of treatment.

### Efficacy

**Fig. 1:**
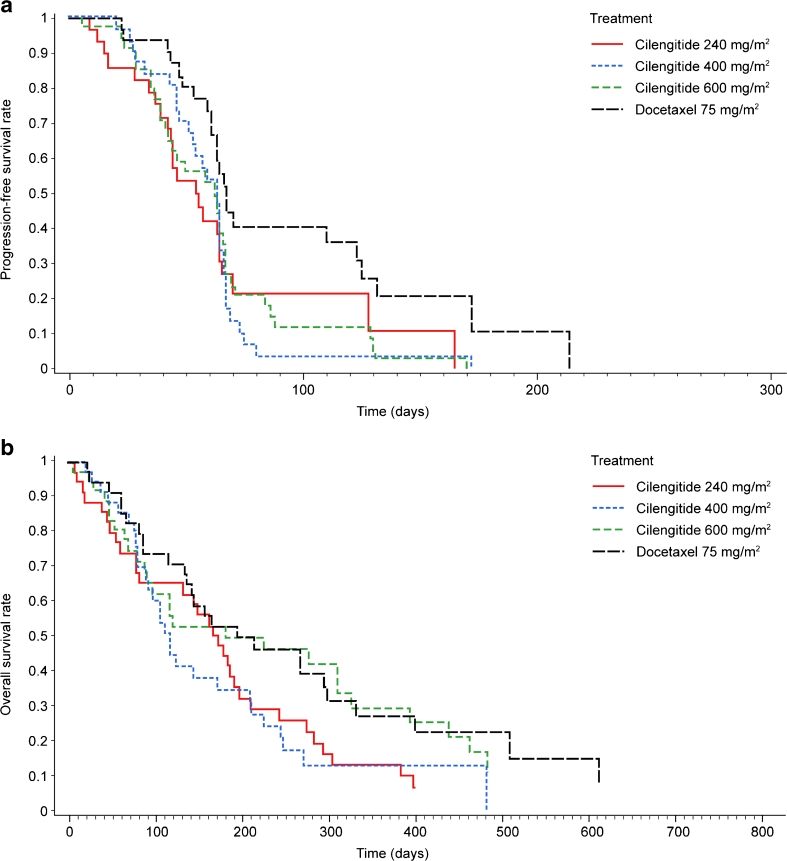
Progression-free survival (**a**) and overall survival (**b**) of non-small-cell lung cancer patients treated with cilengitide 240, 400, or 600 mg/m^2^, or docetaxel 75 mg/m^2^

Median PFS (Table 2, Figure 1a) was longer with cilengitide 400 and 600 mg/m^2^ (63 days) than with cilengitide 240 mg/m^2^ (54 days, 95 % CI 43–64) and similar to that with docetaxel (67 days, 95 % CI 61–123). Median OS (Table 2, Figure 1b) was shorter with cilengitide 400 mg/m^2^ (117 days) than with cilengitide 240 mg/m^2^ (173 days, 95 % CI 81–197) or 600 mg/m^2^ (181 days, 95 % CI 90–326), or docetaxel (194 days, 95 % CI 135–298). Median OS was similar for cilengitide 600 mg/m^2^ and docetaxel. The cilengitide 600 mg/m^2^ and docetaxel arms had similar 1-year survival rates: 29 % and 27 %, respectively (Table 2). No patient had a CR, and only five patients (all in the docetaxel group) were reported as having a PR (Table 3). In two of these five cases, responses were not confirmed according to RECIST. SD was also achieved by more patients in the docetaxel group than in the cilengitide groups (Table 3).

**Table 2:** Primary and secondary outcome measures of survival in intention-to-treat population

Outcome	Cilengitide 240 mg/m^2^ *n* = 35	Cilengitide 400 mg/m^2^ *n* = 35	Cilengitide 600 mg/m^2^ *n* = 36	Docetaxel 75 mg/m^2^ *n* = 34
Median PFS, days [95 % CI]	54 [43–64]	63 [53–66]	63 [42–67]	67 [61–123]
Median OS, days [95 % CI]	173 [81–197]	117 [92–209]	181 [90–326]	194 [135–298]
1-year survival rate, % [95 % CI]	13 [1.2–24.4]	13 [0.4–25.5]	29 [12.3–46.5]	27 [10.4–43.4]

CI, confidence interval; OS, overall survival; PFS, progression-free survival

**Table 3:** Tumor response rates of intention-to-treat population

Response	Cilengitide 240 mg/m^2^ *n* = 35	Cilengitide 400 mg/m^2^ *n* = 35	Cilengitide 600 mg/m^2^ *n* = 36	Docetaxel 75 mg/m^2^ *n* = 34
Complete response, *n*	0	0	0	0
Partial response, *n* (%)	0	0	0	5^a^ (15)
Stable disease, *n* (%)	7 (20)	3 (9)	7 (19)	11 (32)
Progressive disease, *n* (%)	17 (49)	26 (74)	23 (64)	12 (35)
Response rate, %	0	0	0	15
Tumor growth control, %	20	9	19	47

^a^Two of these five partial responses were not confirmed according to Response Evaluation Criteria In Solid Tumors (RECIST)

### Safety

AEs of any degree of severity were experienced by 98 % of patients. Grade 3/4 treatment-related AEs were more common among docetaxel-treated patients (Table 4): 13 (41 %) experienced more than one AE, compared with two patients (6 %) in the 240 mg cilengitide arm and four patients (11 %) in each cilengitide group receiving the higher doses (Table 4). The incidence of grade 3/4 nausea and fatigue was comparable across treatment arms, but hematologic toxicity was more common with docetaxel. Table 5 shows the frequency of grade 3/4 treatment-emergent AEs regardless of the relationship to the investigational agents. Sixteen percent of docetaxel-treated patients experienced grade 3/4 leukopenia and neutropenia. Hematologic toxicity of this severity did not occur with cilengitide.

**Table 4:** Grade 3/4 treatment-related adverse events

Adverse event^a^, *n* (%)	Cilengitide 240 mg/m^2^ *n* = 34^b^	Cilengitide 400 mg/m^2^ *n* = 35	Cilengitide 600 mg/m^2^ *n* = 36	Docetaxel 75 mg/m^2^ *n* = 32^c^
Patients with ≥1 adverse event, *n* (%)	2 (6)	4 (11)	4 (11)	13 (41)
Nausea	1 (3)	0	0	2 (6)
Chest pain	0	2 (6)	0	0
Dyspnea	1 (3)	1 (3)	0	1 (3)
Leukopenia	0	0	0	4 (13)
Neutropenia	0	0	0	4 (13)
Fatigue	0	1 (3)	1 (3)	1 (3)

^a^Unless otherwise stated, grade 3/4 treatment-related AEs occurring in two or more patients in any treatment group

^b^One patient did not receive study drug

^c^Two patients did not receive study drug

**Table 5:** Grade 3/4 treatment-emergent adverse events^a^

Adverse event (preferred term)	Cilengitide 240 mg/m^2^ *n* = 34	Cilengitide 400 mg/m^2^ *n* = 35	Cilengitide 600 mg/m^2^ *n* = 36	Docetaxel 75 mg/m^2^ *n* = 32
Patients with any AE, *n* (%)	21 (61.8)	26 (74.3)	27 (75.0)	24 (75.0)
Dyspnea	8 (23.5)	10 (28.6)	12 (33.3)	5 (15.6)
Asthenia	1 (2.9)	1 (2.9)	0	5 (15.6)
Neutropenia	0	0	0	5 (15.6)
Leukopenia	2 (5.9)	0	0	4 (12.5)
Pneumonia	1 (2.9)	0	1 (2.8)	4 (12.5)
Tumor pain	0	2 (5.7)	2 (5.6)	1 (3.1)
Chest pain	2 (5.9)	4 (11.4)	1 (2.8)	0
Pleural effusion	2 (5.9)	2 (5.7)	2 (5.6)	0
Back pain	3 (8.8)	3 (8.6)	1 (2.8)	0

^a^These data were re-analyzed in March 2011 according to MedDRA version 10.0. Adverse events (AEs) are ordered by frequency of occurrence in the docetaxel group

Fifteen patients died during the study due to progressive disease, pneumonia, dysuria, dyspnea, worsening of chronic obstructive pulmonary disease, or thrombocytopenia (three, five, and four patients in the cilengitide 240, 400, and 600 mg/m^2^ groups, respectively, and three patients in the docetaxel group). Only two deaths were classified as related to treatment. One was a case of tumor progression (in the cilengitide 240 mg/m^2^ arm) and one a case of thrombocytopenia (in a patient treated with 600 mg/m^2^ cilengitide).

There were generally no clinically significant differences between the treatment groups with respect to vital signs (blood pressure and heart rate), ECG findings, or laboratory assessments. A higher proportion of docetaxel-treated patients (19 %) had abnormal neutrophil counts compared with cilengitide-treated patients (9 %, 3 %, and 14 %, in the 240, 400, and 600 mg/m^2^ groups, respectively).

## Discussion

Integrin inhibitors are a novel class of anticancer agents, being developed in response to the continuing need for therapies that target different components of the tumorigenic process [10]. Integrins enable binding between tumor cells and the extracellular matrix, and integrin signaling regulates tumor cell migration, invasion, proliferation, and survival. Integrins are also involved in angiogenesis.

Surgical carcinoma specimens show expression of αVβ3 and αVβ5 integrins by tumor and stromal cells and in the vasculature of lung tumors [20], confirming earlier evidence that integrin inhibition is a rational therapeutic strategy in NSCLC [21]. PRs to single-agent cilengitide have been reported in phase I and II studies in patients with glioblastoma, where cilengitide has been more extensively studied [22, 23].

This phase II trial was the first to assess the efficacy, safety, and tolerability of cilengitide in the treatment of NSCLC. Median PFS at higher doses of single-agent cilengitide (400 and 600 mg/m^2^) was similar to that with single-agent docetaxel, which remains a standard of care and a relevant comparator in clinical trials [8]. One-year survival rates were similar for cilengitide 600 mg/m^2^ (29 %) and docetaxel (27 %). So too was median OS (194 days for docetaxel and 181 days for cilengitide 600 mg/m^2^). Interestingly – and supporting the relevance of this study – the median 194-day OS we report with 3-weekly docetaxel closely matches the median 189-day OS in a meta-analysis of data from five second-line docetaxel trials involving 433 patients [24].

No CRs were seen with either cilengitide or docetaxel. The PR rate with docetaxel was 15 % while no cilengitide-treated patient had a PR. The PR rate with docetaxel seen in this study is similar to the 13–22 % rates reported in other monotherapy studies of the taxane in advanced NSCLC [25–27].

Although the median PFS of single-agent cilengitide 600 mg/m^2^ was similar to that of docetaxel, there were important differences in the toxicity profiles of the two agents. The safety profile of cilengitide in terms of grade 3/4 treatment-related AEs was superior to that of docetaxel: while 16 % of docetaxel-treated patients experienced grade 3/4 leukopenia and neutropenia, no such cases were reported in cilengitide-treated patients. Indeed, cilengitide was well tolerated generally in the present trial, and this has remained the case even at the substantially higher doses used in subsequent studies, such as those in which patients with recurrent glioblastoma received up to 2400 mg/m^2^ of the drug [23, 28]. Importantly, the AEs with cilengitide showed little overlap with those of docetaxel, suggesting that the two agents might be combined, either simultaneously or sequentially.

This study was conducted 10 years ago, when requirements for histology were less rigorous than today, and trials in non-selected populations of NSCLC patients were routine. The direct applicability of its results to current management is therefore limited. However, the study remains important in showing the potential of cilengitide as a well-tolerated treatment option in advanced NSCLC and supports its development as a combination partner in therapy. An ongoing phase II study (CERTO) is investigating cilengitide in combination with platinum-based chemotherapy and the growth factor receptor inhibitor cetuximab as first-line treatment for advanced NSCLC. Recent results from the safety run-in phase of this trial have shown that cilengitide (at doses of 1000 or 2000 mg twice weekly) combined with standard therapy was well tolerated with no unexpected AEs and no dose-limiting toxicities [29].
